# Prognostic Value of Asymmetric Dimethylarginine in Patients with Heart Failure: A Systematic Review and Meta-analysis

**DOI:** 10.1155/2020/6960107

**Published:** 2020-07-04

**Authors:** Wenjun Pan, Baotao Lian, Haining Lu, Pengda Liao, Liheng Guo, Minzhou Zhang

**Affiliations:** ^1^Guangzhou University of Chinese Medicine, Guangzhou 510405, China; ^2^The Second Clinical College of Guangzhou University of Chinese Medicine, Guangzhou 510120, China; ^3^Guangdong Provincial Hospital of Chinese Medicine, Guangzhou 510120, China

## Abstract

**Objective:**

Asymmetric dimethylarginine (ADMA), an endogenous inhibitor of nitric oxide (NO) synthesis, is reported to be a risk factor for cardiovascular disease. The purpose of the present study is to investigate whether ADMA is an independent predictor for future mortality and adverse clinical events among patients with heart failure (HF).

**Methods:**

Electronic literature databases (Central, MEDLINE, and Embase) were searched for relevant observational studies on the prognostic value of ADMA in HF patients published before January 2019. Pooled hazard ratios (HRs) or odds ratio and the corresponding 95% confidence interval (CI) were calculated for risk evaluation.

**Results:**

10 studies with 2195 participants were identified and analyzed. The pooled HR of composite clinical events for the highest vs. lowest quartiles from categorical variable results was 1.34 (95% CI: 1.15-1.57, *P* < 0.001, *I*^2^ = 0%), which is 1.31 (95% CI: 1.10-1.55, *P* < 0.005, *I*^2^ = 0%) in the subgroup of acute decompensated HF. The pooled HR of composite clinical events from continuous variable results was 1.41 (95% CI: 1.21-1.63, *P* < 0.001, *I*^2^ = 21.9%), with 0.1 *μ*M increment accounting for the increasing 25% risk for composite adverse clinical events. The pooled HR for all-cause mortality was 2.38 (95% CI: 1.48-3.82, *P* < 0.001, *I*^2^ = 0%) after sensitivity analysis. Two studies reporting the HR of inhospital mortality in HF patients regarded it as a prognostic indicator, with categorical variable HR as 1.26 (95% CI: 1.07-1.84, *P* < 0.05) and continuous variable OR as 2.15 (95% CI: 1.17–4.29, *P* < 0.05).

**Conclusions:**

ADMA is an independent predictor for composite clinical outcomes among HF patients with both short-term and long-term prognostic value.

## 1. Introduction

Heart failure (HF) is on the rise of the leading cause of death and hospitalization worldwide. Accurate prognostic biomarkers are crucial for risk assessment and timely and appropriate therapeutic interventions of HF [1]. Brain natriuretic peptide (BNP) has been used successfully to identify HF in patients admitted for dyspnea and helps for predicting the prognosis of patients with HF [2, 3]. However, BNP or N-terminal probrain natriuretic peptide- (NT-pro-BNP-) guided HF therapy is of uncertain benefit and cannot be universally advised [2, 3]. Thus, increased BNP concentrations often require additional biomarker tests.

Asymmetric dimethylarginine (ADMA) is an endogenous inhibitor of nitric oxide (NO) synthesis [4], which is the risk factor of cardiovascular disease including coronary heart disease, hypertension, stroke, and atrial fibrillation (AF) [5–7]. Besides, ADMA was reported to be a predictor for major adverse cardiovascular events and the mortality in AF and acute myocardial infarction patients [8, 9]. In recent years, a strong relationship between ADMA and HF has been demonstrated. ADMA is negatively correlated with left ventricular ejection fraction and positively correlated with New York Heart Association cardiac functional grading, BNP levels, and the incidence of HF [10–12]. As compared to healthy controls, the plasma level of ADMA was found to significantly increase in HF patients. In addition, higher ADMA levels might confer to severe and exacerbated HF [13].

However, the prognostic value of ADMA in HF patients has not been fully elucidated. Some clinical findings suggested ADMA as a strong predictor for the prognosis of HF [14–16], while others implied ADMA was not associated with an elevated mortality risk in HF [17]. In this study, we aim to provide a systematic review and meta-analysis to study the prognostic value of ADMA in HF patients.

## 2. Methods

### 2.1. Search Strategy

We performed an electronic literature search of MEDLINE through PubMed, Central through Cochrane Library, and Embase for relevant observational studies assessing the association between ADMA levels and HF with no language restriction. The search time was from the beginning of data inception until May 2018. The searched terms were expressed in Boolean combinations: (heart failure or cardiac failure or myocardial failure or heart decompensation) and (asymmetric dimethylarginine or ADMA) in the combination of medical subject heading (MeSH) and free text. Bibliographies of the included studies were searched as well to avoid missing relevant articles.

### 2.2. Selection of Studies

Studies were eligible for the systematic review if they met the following criteria: (1) prospective cohort or retrospective studies, (2) HF was defined as patients with typical signs and symptoms of HF and New York Heart Association (NYHA) functional classification II–IV, (3) described the association between ADMA levels and the prognosis of HF, (4) the outcomes of interests were death and rehospitalization, and (5) reported unadjusted or adjusted risk estimates for the outcomes, such as RR or HR with a 95% CI. The studies investigating the prognosis of patients accompanied by other diseases such as acute coronary syndrome, renal dysfunction, inflammatory diseases, and systematic diseases or younger than 18-year-old were excluded.

### 2.3. Quality Assessment

Two authors (Wenjun Pan and Baotao Lian) independently assessed the quality of all included studies by using the Newcastle-Ottawa Scale. This scale awards a maximum of nine stars over three categories: selection (4 stars), comparability (2 stars), and outcome (3 stars), with higher quality studies achieving a greater number of stars. For some discrepancies, we discussed with Dr. Minzhou Zhang and Dr. Liheng Guo, as well as consulted the writers for research details.

### 2.4. Data Extraction

Data extraction of all included studies was performed by two authors (Wenjun Pan and Baotao Lian), respectively, via a preset standardized electrical form. The following characteristics were extracted: authors, publication year, patients' baseline status, study design, study sample size, gender, length of follow-up, adjusted variables, adjusted or non-adjusted HR for mortality, and 95% CI.

### 2.5. Statistical Analysis

The unadjusted and multivariable-adjusted HRs or OR for categories (highest versus lowest categories) reported in the original articles were used to estimate the associations between ADMA levels and HF. We calculated the unadjusted HR using original data published in the studies if relevant effect sizes were not available. Forest plots were used to evaluate HRs and corresponding 95% CIs for included studies. Overall HRs were calculated using random-effects models (Der Simonian and Laird). Potential heterogeneity among studies was evaluated using the *I*^2^ statistic, which is a quantitative measure of inconsistency across studies. If there is significant heterogeneity (*I*^2^ > 50%, *P* < 0.1), we conducted the sensitivity analysis to test the source of heterogeneity. A two-tailed *P* value below 0.05 was considered statistically significant. All statistical analyses were performed using Stata 12.0.

## 3. Results

### 3.1. Search Results

**Figure 1:**
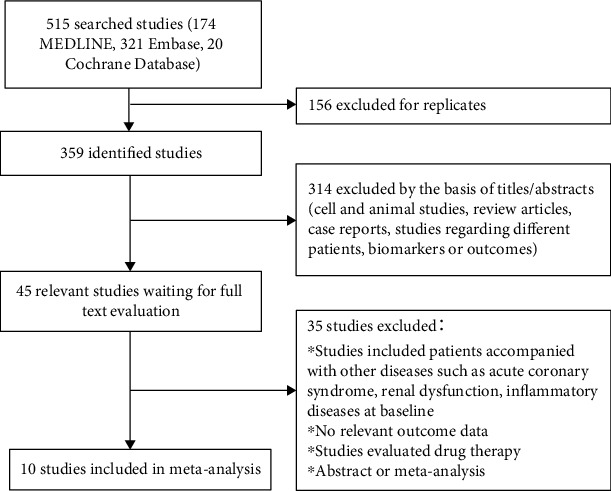
Flowchart of study search and identification.

The search process is summarized in Figure 1. The initial search yielded 515 results, of which 156 articles were excluded for duplicates. After screening the title or abstract, 314 studies were eliminated, and the remaining 45 articles were further identified by reading the full text. According to the predefined inclusion criteria, ten studies enrolling 2195 participants from 2007 to 2018 were included in the meta-analysis [14–22].

### 3.2. Study Characteristics

As demonstrated in Table 1, among the 10 studies, 7 studies considered ADMA as a continuous variable while 4 studies as categorical variable (with one study reported both results). Three studies assessed the impact of the ADMA level on the incidence of major adverse cardiovascular events (MACEs), and 8 studies evaluated the effects of ADMA level on all-cause mortality, with 6 studies regarding cardiovascular events including HF rehospitalization and cardiac transplantation. The duration of follow-up varied from 3 months to 73.2 months. All the patients were older than 18 year old, with median age ranging from 52.7 to 75.2 years old. Seven studies adjusted the HRs or OR to confounders such as age, sex, left ventricular ejection fraction (LVEF), and glomerular filtration rate (GFR). According to the Newcastle-Ottawa Scale criteria, 7 studies are of high quality, while the other 3 studies had suboptimal quality.

### 3.3. ADMA and Prognosis of HF

**Figure 2:**
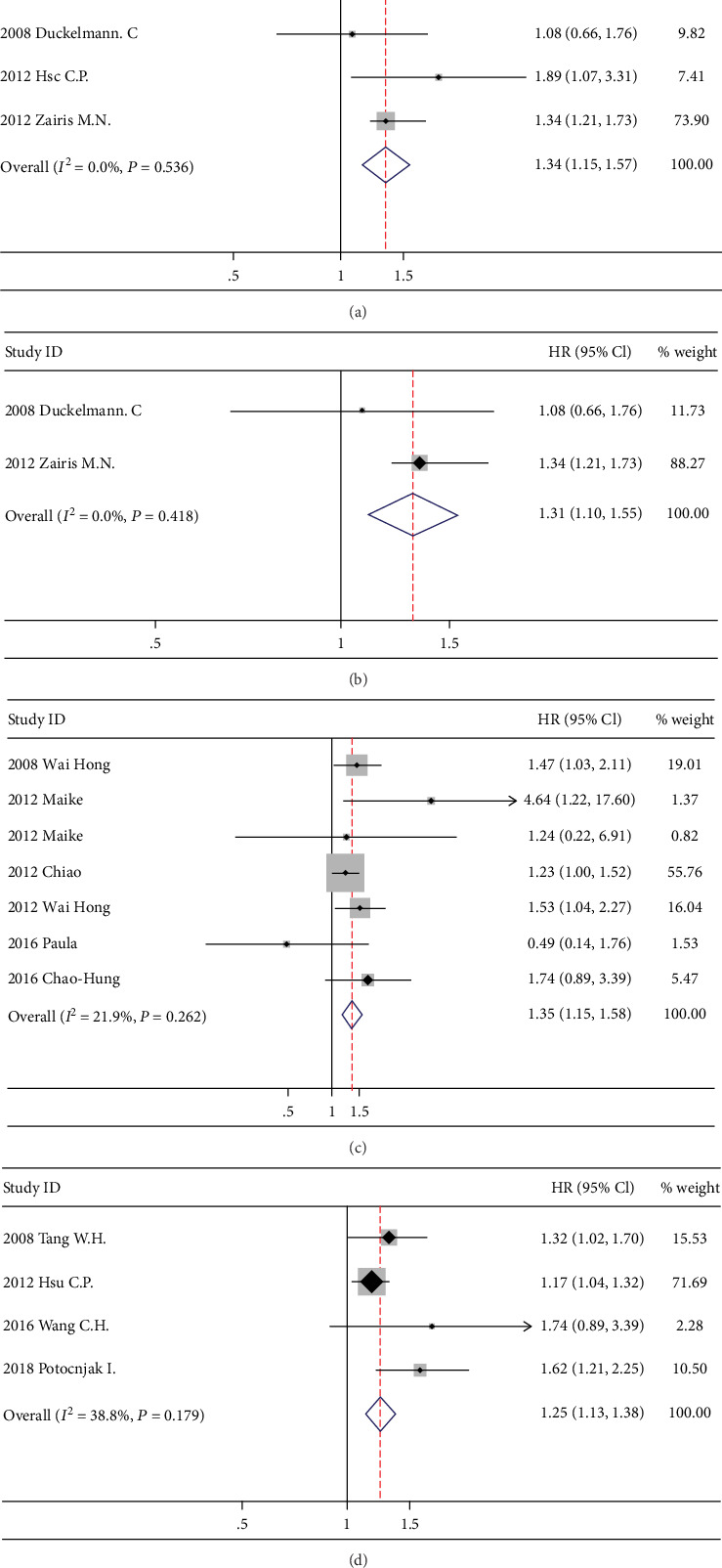
Forest plot of the hazard ratio of ADMA for a composite adverse cardiovascular event.

Random-effects model meta-analysis showed that the increased level of ADMA was significantly associated with adverse cardiovascular events including death, cardiac decompensation, and other major adverse cardiovascular events. The pooled HR for the 4 studies which considered ADMA as a categorical variable is 1.34 (95% CI: 1.15-1.57, *P* < 0.001, *I*^2^ = 0%) (Figure 2(a)). The pooled HRs for the subgroup of acute decompensated HF is 1.31 (95% CI: 1.10-1.55, *P* < 0.005, *I*^2^ = 0%) (Figure 2(b)). The pooled HR for the 5 studies which considered ADMA as a continuous variable is 1.35 (95% CI: 1.15-1.58, *P* < 0.001, *I*^2^ = 21.9%) (Figure 2(c)). As shown in Figure 2(d), the pooled HR for the 4 studies which reported ADMA as a continuous variable in a per 1 unit or 1 SD manner is 1.25 (95% CI: 1.13-1.38, *P* < 0.001, *I*^2^ = 38.8%). There was no significant heterogeneity among both categorical value studies and continuous value studies (*I*^2^ = 0, *P* = 0.536; *I*^2^ = 21.9%, *P* = 0.262; and *I*^2^ = 38.8%, *P* = 0.179, respectively) (Figure 2).

**Figure 3:**
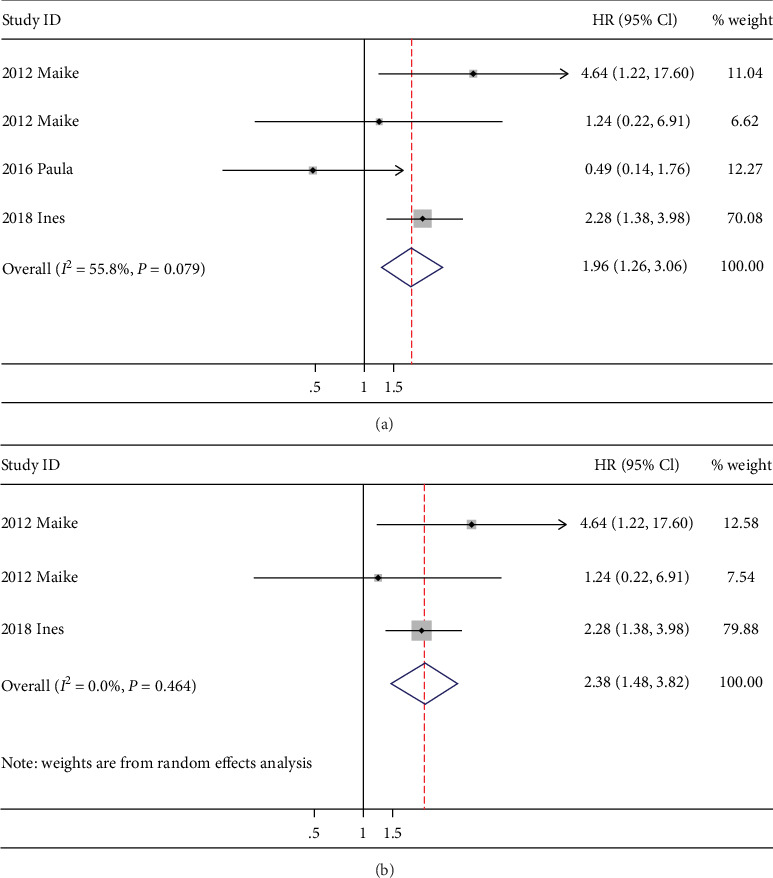
Forest plot of the hazard ratio of ADMA for all-cause mortality.

Five studies explored the prognostic value of ADMA on the all-cause mortality of HF patients. The pooled HR of continuous variable results reporting all-cause mortality is 1.96 (95% CI: 1.26-3.06, *P* < 0.01, *I*^2^ = 55.8%) (Figure 3(a)), which supports ADMA as a vital biomarker evaluating HF mortality. As there is significant heterogeneity, we conducted sensitivity analysis. The pooled HR for all-cause mortality is 2.38 (95% CI: 1.48-3.82, *P* < 0.001, *I*^2^ = 0%) after excluding the study causing the heterogeneity (Figure 3(b)). Two studies reported the inhospital mortality of HF patients. Although reported in different ways, both of them regarded ADMA as a prognostic indicator with HR of highest quartile vs. lowest quartile as 1.26 (95% CI: 1.07-1.84) and continuous variable OR as 2.15 (95% CI: 1.17–4.29), demonstrating its value in assessing the short-term prognosis in HF patients.

## 4. Discussion

Our meta-analysis of 10 relevant studies involving a total of 2195 participants confirms that ADMA is significantly associated with the risk of death in HF patients in both the short-term and the long-term context, suggesting its prognostic utility in the setting of HF. Although there were diverse statistical methods for calculating the HRs of ADMA, the general heterogeneity was comparably low among the studies. The detection of serum ADMA levels would provide a valid prediction for the prognosis of HF patients.

In our results, we found that ADMA was associated with increased adverse cardiovascular events in HF patients no matter if it was considered a categorical variable or a continuous variable. In the categorical variable studies, the inclusion criteria of was LVEF less than 45%. Patients with increased ADMA values had 34% higher incidence of adverse cardiovascular events, which indicated the prognostic value of ADMA in HF with reduced EF (HFrEF) patients. Firstly, ADMA might confer to low cardiac output. As reported by Achan et al., intravenous low-dose ADMA injection in healthy volunteers produced several adverse cardiovascular effects, including reduced heart rate and cardiac output as well as increased mean blood pressure [23]. As a NOS inhibitor, the augmented ADMA would reduce the bioactivity of NOS, inhibits the downstream S-nitrosylation and activation of the cardiomyocyte ryanodine receptor, resulting in the disturbance of cytosolic Ca^2+^ release and cardiac contractility [24].On the other hand, if renal perfusion is reduced in HFrEF, ADMA might increase because ADMA is excreted by the kidneys [25]. So there is a vicious circle between increased ADMA levels and the progress of HF. However, when we did data extraction, we used RRs adjusted for glomerular filtration rate to avoid the interference of renal function as much as possible.

In patients with acute decompensated HF, higher ADMA levels were associated with 31% increment of adverse cardiovascular events. The presence of congestion might influence the ADMA values. In an ARISTOTLE substudy, ADMA was found to increase with the presence of congestive heart failure in atrial fibrillation patients [8]. Masayuki et al. found that compared to chronic compensated HF patients, ADMA was significantly higher in patients with acutely exacerbated chronic HF [13]. It was reported that in end-stage failing hearts, left ventricular assist devices helped to increase the expression of ADMA-degrading enzyme, dimethylaminohydrolases-1 (DDAH-1), suggesting that congestion might account for the increased ADMA values owing to less degradation by DDAH-1 [26]. In our meta-analysis, we included RRs adjusted for BNP and EF if they are available to rule out the interference of congestion for ADMA's prognostic power.

According to our results, ADMA is associated with increased all-cause mortality as well as inhospital mortality in patients with HF. Although there was a remarkable heterogeneity among the included studies regarding all-cause mortality, the association persisted after excluding the study accounting for the obvious heterogeneity. It was hard to avoid the heterogeneity in observation studies as the HRs were adjusted for different confounders, and patients were with different primary diseases and medical history.

It remains controversial if drug therapy affects ADMA levels. Although several angiotensin-converting enzyme inhibitors (ACEIs), angiotensin II-receptor blockers (ARBs), statins, and beta blockers are proven to decrease ADMA levels after long-term treatment [27–29], it showed no change in atorvastatin after 6 weeks of therapy in nonischemic heart failure patients [30]. It is intriguing that after pharmacological treatment with diuretics, digoxin, ACEIs, or ARBs, ADMA levels were increased compared to baseline values (pretreatment) in acute congestive HF patients [31]. However, as higher ADMA levels are associated with several cardiovascular disorders, ADMA-targeted therapy is still a promising strategy for cardiovascular diseases. L-Arginine infusion could compete with ADMA for NO synthesis [32], and 5-methyltetrahydrofolate could increase DDAH activity and decrease ADMA levels [33]. Both of them showed endothelial protective effects in HF patients. ADMA might be a potential target in HF treatment.

ADMA is a global NOS inhibitor, which could inhibit the bioavailability of NOS1 (neuronal NOS (nNOS)), NOS2 (inducible NOS (iNOS)), and NOS3 (endothelial NOS (eNOS)) [34]. ADMA inhibits NO production by competing with arginine for NOS binding. Firstly, it would deteroriate NO-dependent vasodilation therefore impairing relaxation of coronary arteries [35]. Secondly, ADMA could diminish the S-nitrosylation of proteins in cardiomyocytes, which could cause calcium overload if it disturbs posttranslational modification of ryanodine receptors and L-type calcium channels [24]. Furthermore, the NO-cGMP-PKG signaling pathway targets several proteins involved in cardiac contractility and hypertrophy [36]. ADMA, however, would facilitate cardiac constrictive disorder and hypertrophy in HF via the inhibition of NO production.

Evaluation of ADMA levels is helpful in clinical practice. ADMA was demonstrated to help risk stratification of high-risk patients as compared to NT-pro BNP alone. Duckelmann et al. found that patients with ADMA and NT-pro BNP concentrations in the highest tertile had a 4.5-fold higher risk (95% CI 2.1 to 9.7; *P* < 0.001) for a clinical endpoint compared with subjects without ADMA > 0.64 mol/L or NT − pro BNP > 2512 pg/mL after 6 months follow-up [18], suggesting a better risk stratification with the combination of NT-pro BNP and ADMA in HF patient. ADMA-targeted therapy might be a promising strategy in HF treatment. Besides the conventional drugs for HF like ACEIs, ARBs and beta-blockers, both arginine and citrulline could decrease ADMA values and suppress ventricular remodeling in clinical trials [32, 37], implicating them as salutary supplements in improving the prognosis of HF patients.

## 5. Limitations

We did not have patient-level data and were unable to identify optimal cutpoint for ADMA levels in prognostic scenarios. In studies of categorical ADMA, we only included the highest and lowest quantiles, which might exaggerate its prognostic value. As all included studies were observational studies, confounders cannot be entirely eliminated. Besides, the risk factors and statistical model for which each HR or OR adjusted might vary from one another, so we were not able to account for all HF risk factors in this meta-analysis. Thus, the result should be interpreted with caution in clinical practice. There might be some systematic errors during the detection of ADMA by HPLC in different studies. A commercial enzyme-linked immunosorbent assay kit might be a good substitution because of its simple and high-throughput properties [38], which will facilitate ADMA detection in the clinic.

## 6. Conclusion

This systematic review and meta-analysis have demonstrated the prognostic utility of ADMA in patients with HF. The increased levels of ADMA were significantly associated with higher risk of mortality and other adverse events in HF patients. Baseline ADMA levels may be a practical predictor of both inhospital mortality and long-term death in patients with HF.

## Figures and Tables

**Table 1:** Characteristics of included studies.

First author	Publication year	Patients	Sample size	Mean/median age (years)	ADMA assay	Variable type	Comparison	Mean follow-up (months)	Variables adjusted	Outcomes	NOS
Duckelmann, C.	2007	CHF	276	70	HPLC	Categorical	Highest tertile vs. lowest tertile > 0.64 vs. <0.51 *μ*mol/L	14.2	Age, sex, GFR, LVEF, systolic blood pressure, and BMI	Cardiac decompensation, MACE, or all-cause death	9/9
Duckelmann, C.	2008	Acute decompensated HF	118	73	HPLC	Categorical	Above median vs. below median > 3051 vs. <3051 pg/mL	10.7	None	MACE, or all-cause death	9/9
Tang, W. H.	2008	Stable but symptomatic HF	132	57.8	HPLC	Continuous	Per ADMA log-1 SD increment	33	Age, eGFR, and LVEF	Death, cardiac transplantation, or heart failure hospitalization	8/9
Anderssohn, M	2012	CHF due to ICM or DCM	341	55.1	HPLC	Continuous	Per ADMA log-1 unit increment	39.6	None	All-cause mortality	8/9
Hsu, C. P.	2012	CHF	285	70	HPLC	Continuous & categorical	Above vs. below the best discriminating ADMA level by ROC curve > 0.48 vs. <0.48 mmol/L	26.4	Age, log NT-proBNP level, eGFR and LVEF	Major adverse cardiovascular events (MACE) or cardiac decompensation needing hospitalization	9/9
Zairis, M. N.	2012	Acute decompensation CHF	651	73	HPLC	Categorical	Highest quartile vs. lowest quartile 1.83~3.70 vs. 0.21~0.76 *μ*mol/L	12	Age, sex, SBP, HR, atrial fibrillation, acute pulmonary edema, LVEF, eGFR, Na, and BNP	Cardiac mortality	9/9
Mommersteeg, P. M. C.	2016	HF	104	66	UPLC	Continuous	Ln ADMA	73.2	Age, sex, poor exercise tolerance [6MWT<300 m] and comorbidity burden	All-cause mortality	9/9
Wang, C. H.	2016	AHF or decompensated CHF	136	58.9	UPLC	Continuous	ADMA	27.6	Age, LVEF, diabetes mellitus, eGFR, and BNP	HF-related re-ospitalization and all-cause death	9/9
Potočnjak, I.	2018	AHF	152	75.2	HPLC	Continuous	Per ADMA 1 SD increment	3 months	Age, sex, NT-pro BNP, GFR, MAP, and LDL-C	All-cause death	9/9

HPLC: high-performance liquid chromatography; UPLC: ultraperformance liquid chromatography; CHF: chronic heart failure; AHF: acute heart failure; LVEF: left ventricular ejection fraction; ICM: ischemic cardiomyopathy; DCM: dilated cardiomyopathy; eGFR: estimated glomerular filtration rate; NOS: Newcastle-Ottawa Scale criteria; ROC: receiver operating characteristic; 6MWT: Six-Minute Walk Test.
